# The “Empty-Goal” Rule Change from the Perspective of International-Level Team Handball Goalkeepers

**DOI:** 10.3390/ijerph19116506

**Published:** 2022-05-26

**Authors:** Reuven Iusepolsky, Elia Morgulev, Sima Zach

**Affiliations:** 1 The Academic College at Wingate, Wingate Institute, Netanya 4290200, Israel; 1reuvenaa@gmail.com (R.I.); simaz@wincol.ac.il (S.Z.); 2 Israel Handball Federation, Tel Aviv 6948206, Israel; 3 Department of Physical Education, Kaye Academic College of Education, Beer-Sheva 8414201, Israel; 4 Department of Business Administration, Guilford Glazer Faculty of Business and Management, Ben-Gurion University of the Negev, Beer-Sheva 8410501, Israel

**Keywords:** handball, goalkeeper, empty goal, rules, practice

## Abstract

The 2016 “empty-goal” rule change in team handball allowed for swift goalkeeper-player substitutions, which opened the door to a variety of tactical solutions that could not be implemented prior to the change. This change is one of many rule changes that have taken place in ball games in general and in handball in particular that were aimed to improve the competition and make gameplay more interesting. Previous literature shows that more often than not, such rule changes have led to unforeseen and undesired effects on players’ and teams’ behavior and performance. The aim of the current study was to consider the empty-goal rule from the goalkeeper’s perspective, as their offense–defense game routine was drastically transformed following the introduction of this new rule. Results of a survey among 95 professional goalkeepers, 80 of whom participated in international matches, revealed that the keepers’ level of confidence in empty-goal situations is moderate to high, that empty goal is rarely practiced more than once a week, and that less experienced goalkeepers are more positive regarding this rule change. Additionally, we found that the amount of empty-goal practice is positively related to the approval of the empty-goal rule among goalkeepers.

## 1. Introduction

At first glance, it may seem that rules for a given sport remain relatively static, with changes only rarely being made [1]. However, a more nuanced examination reveals that in recent decades sport governing bodies have initiated many changes in the way competitions are organized and how sports are played [2]. For instance, in 1999, to make the duration of volleyball and beach volleyball matches more predictable in terms of their length, the rule that only the serving player can score a point was changed, and now, a point is scored from every rally by whichever side wins the rally [3].

In football (soccer), the established rule was to give two points for a win and one for a draw, but then, during the 1980s and 1990s, many national football federations decided to give three points for a win in order to increase the incentive for a more attacking style aimed at winning [4]. In the 1950s, both the National Basketball Association (NBA) and the International Basketball Federation (FIBA) identified the problem of leading teams passing the ball incessantly solely to run out the clock. To address this issue, increase scoring, and make the game more attractive to spectators, a 24 s shot clock was introduces, which limits the time a team may possess the ball before attempting to score [5].

In this vein, the International Handball Federation (IHF) was also faced with the issue of teams attacking very slowly without any meaningful attempts in the direction of the goal. This tactic was being employed by leading teams during the final minutes of a match or by teams playing in numerical inferiority during 2 min suspensions [6]. However, instead of limiting possession with the aid of a shot clock, the IHF delegated the responsibility of preventing unattractive methods of play and intentional delays in the game to the referees. For decades, handball referees raised their hand to forewarn the attacking team about passive playing and subjectively called an offensive game violation in cases where the forewarned team failed to throw at the goal after being warned [7].

In 2016, the IHF introduced a set of rule changes, among which was the new passive play rule aimed at objectifying the passive play decision, as well as the “empty-goal” rule, which we will discuss later. The IHF accompanied these changes with a statement that it was seeking to make the sport of handball even more attractive to spectators with this new set of rules [8].

However, according to the literature, rule changes aimed at making a tournament more competitive or a game more exciting have often led to an unexpected or even undesired occurrence [1]. For instance, the NBA compensates poorly performing teams with better draft selections from the pool of amateur talent in order to sustain a high level of competitive balance in the league; however, the revealed propensity of teams that are out of playoff contention to lose deliberately in order to obtain higher-quality draft selections was unintended by the policy makers [9]. The finding that most defender falls in offensive foul situations are intentional and deceptive acts aimed to mislead the referee (i.e., a flop) is another example of the abuse of basketball rules [10]. Similarly, in athletics, sprinters have been false starting deliberately with an eye to psych out their fellow competitors [1].

In football (soccer), Brocas and Carrillo [11] used game theory to demonstrate how the three-point rule may act as an incentive to make teams play more defensively when they are leading. Dewenter and Namini [12] empirically verified that the three-point reward scheme does not necessarily induce teams to play more offensively, as the rule change has led to a reduction in the number of home-team goals in Germany. Returning to handball, it was argued by Haugen and Guvåg [8] that the 2016 set of rule changes may have actually hindered the uncertainty of outcome (i.e., competitive balance) in European national handball leagues games.

One particularly interesting rule change introduced in 2016 is the “empty-goal” rule, which allowed for swift substitution of the goalkeeper for another court player in an attack without the player wearing a special shirt (as was the case before the change). This change made it possible for the coach to leave the goal empty and bring an extra court player to the offensive end when in numerical equality (seven against six) or to compensate for numerical inferiority due to a 2 min suspension (six against six) [7].

Krahenbühl et al. [13] interviewed four elite-level coaches and concluded that the empty-goal tactic was being used mainly to maintain numerical equality under 2 min suspensions or to reach numerical superiority in decisive moments during the final minutes of close matches. Marczinka and Gál [7] analyzed 15 matches that took place during the 2017 Men’s World Championships in order to explore how often and effectively coaches use the empty goal. Based on this limited sample, the authors indicated that the empty-goal tactic was used in situations of numerical inferiority, on average, twice as much as in situations of numerical superiority and that the coaches pulled the goalkeeper and sent in a court player when losing, on average, twice as often as when winning. Due to the small sample size, the results regarding the effectiveness of this tactic were not decisive in this study.

Gümüş et al. [14] assessed 39 of games in the 2020 Women’s World Championships and showed once again that the empty-goal rule was mainly used by penalized teams to achieve numerical equality (six against six), rather than playing seven against six. As for its effectiveness, the authors stated that the empty-goal rule may yield small gains in scoring on the offensive end, whereas the risk taken by leaving the net empty does not cause any statistically significant harm. In an another study, Gümüş and Gençoğlu [15] analyzed all 62 matches that were played in the Men’s European Handball Championship in 2020. The authors concluded that the losing teams had more missed shots, technical errors, and received goals as a result of using this tactic. However, it was also suggested by the authors that playing with an additional field player could provide an opportunity for advantageous attacking during critical moments of the game and make handball a more attractive team sport in the future.

Krahenbühl et al. [16] analyzed 15 games in the 2017 Women’s Handball World Championship and confirmed that the empty-goal rule is used mainly to offset the numerical imbalance caused by a two-minute suspension. They also reported that there was a significant increase in the number of errors when using the additional field player to gain numerical superiority in attack.

Using big data techniques (see [17]), Neuberg and Thiem [18] were able to assess more than thirty thousand possessions where the empty goal was used to outnumber the opponent and obtain a numerical advantage in an offensive attack. The authors found that underdogs and trailing teams opted for the use of a seventh field player more often than leading teams. The results indicated that near the end of the match, trailing favorites benefit from the use of a seventh field player, whereas no such effect was observed for trailing underdogs. On the other hand, near the end of a match, leading teams, both favorites and underdogs, suffer from the use of this risky tactic.

Whereas the findings concerning the effectiveness of the empty-goal tactic are inconclusive, it seems that the opinions of international-level coaches are rather negative. In a recent survey conducted by the German magazine “Handball Woche” among 39 elite-level coaches, 30 stated that they would prefer to return to the old rule. It was argued, for example, that shots on an empty goal are unattractive to spectators and that the 2 min suspension effect has now been reduced as a result of goalkeeper–player substitutions so that it no longer imposes a meaningful punishment for aggressive and dangerous defense.

In the current study, we aimed to consider the empty-goal situation from the goalkeeper’s perspective. Hansen et al. [19] stressed that goalkeepers play a very important role in handball, and that it is well recognized among coaches that goalkeeper performance can predict team rankings in major tournaments. Kajtna et al. [20] argued that the role of the goalkeeper is particularly exposed, and therefore, keepers are more prone to the effects of pressure. In this context, it is important to realize that until 2016, goalkeepers experienced very few instances of sprinting during games [21], whereas after the 2016 rule change, they found themselves in a new reality in which they were required to perform multiple sprints without getting tired, leaping and running in order to save long-distance shots, making immediate substitutions despite interference from other players, and getting up quickly from the bench to a state of fast running.

Obviously, the goalkeepers had no particular reason to practice these skills until 2016, and one of the objectives of the current study was to determine the extent to which these skills are being practiced today. In this vein, we also aimed to evaluate the level of goalkeepers’ confidence in adapting to a string of physical and mental demands imposed on them by the empty-goal tactic and to assess the amount of stress that empty-goal situations may induce. Finally, we are eager to investigate goalkeepers’ attitude towards the empty-goal rule.

## 2. Methods

### 2.1. Participants

Inclusion criteria were age >18 years old goalkeepers that have played at the highest level of competition in their respective countries after 2016. Participants were 95 professional handball goalkeepers (68 men and 27 women) aged 18–52 from 28 countries. Mean years of playing at a professional level was 8.99 (SD = 6.42). A total of 63 participants were members of their respective national team, 42 had participated in the European Handball Championship or other continental championships, 27 had participated in the World Handball Championship, and 6 had participated in the Olympic Games.

### 2.2. Questionnaire

With the questionnaire (see Supplementary Materials) we aimed to collect data concerning professional goalkeepers’ perceptions of the 2016 empty-goal rule change. The questionnaire was developed by the authors of the study and went through a process of face and content validity using expert examination, comments, and approval, as recommended by others (e.g., [22]). According to this process, a panel of five experts, all professional, international-level handball coaches, examined the clarity of the questionnaire and approved its comprehensiveness and the representativeness of the items with respect to the researched construct.

The questionnaire is comprised of seven parts, as follows: (a) demographic background; (b) confidence in abilities related to the empty-goal rule, consisting of 10 items on a 10-point Likert-type scale (1 = not confident at all; 10 = highly confident), for example: “make a substitution despite the interferences from players on the court” and “contribute to offensive success thanks to my substitution”; (c) advantages and disadvantages of the rule (open-ended questions); (d) anxiety level, consisting of nine items on a five-point Likert-type scale (1 = not anxious at all; 5 = very anxious), for example: “the other goalkeeper on my team is more suitable than I am for this style of play” and “I am replaced, and there is a change in my concentration level due to the frequent entries and exits to and from the court”; (e) sense of belonging and involvement within the team, consisting one question on a 10-point Likert-type scale (1 = not involved at all; 10 = very involved); (f) the number of weekly training sessions to practice the rule; and (g) overall opinion regarding the rule, consisting of one question on a 10-point Likert–type scale (1 = highly against; 10 = highly in favor). Reliability in terms of Cronbach alpha was 0.83 for the 10 confidence items and 0.80 for 9 anxiety items.

### 2.3. Procedure and Data Collection

This study was carried out in accordance with the Code of Ethics of the World Medical Association (Declaration of Helsinki, 7th edition; see [23]). The survey was approved by the Institutional Review Board (IRB) (permission number 301). An electronic version of the questionnaire (Google Forms) was distributed among professional goalkeepers across Europe’s top leagues. We relied on our professional network to recruit 105 respondents who completed the questionnaire; 10 respondents were excluded from the analysis due to missing or erroneous values.

### 2.4. Statistical Analysis

All statistical procedures were calculated using the SPSS statistical package (version 175 20) (IBM, Armonk, NY, USA). Kolmogorov–Smirnov’s test with Lillieforts’s correction was used in order to verify the normality of the data. Levene’s test was used to evaluate the homogeneity of variance of the data. One-way ANOVA was used to compare age, years playing, and years playing at a professional level between the women and men goalkeepers. Chi-square test was used to compare the proportion of women and men who had participated in international matches, the European Handball Federation (EHF) Champions League, a national team, continental championships, and World Championships. A chi-square test was used to assess the correlation between (1) gender and amount of weekly practice of empty-goal scenarios and (2) participation in a national team and the amount of weekly practice of empty-goal scenarios.

Differences in confidence and anxiety by gender, the amount of practice, and participation in a national team were examined using two-way ANOVA and MANOVA analysis with Bonferroni post hoc comparisons. Sense of belonging and overall opinion by gender, the amount of practice, and participation in a national team were analyzed using two-way ANOVA followed by Bonferroni post hoc comparisons.

Multiple linear regression was used as a robustness check, with confidence, anxiety, and overall opinion as response variables.

## 3. Results

### 3.1. Descriptive Statistics and Practice

We open the results section by providing descriptive statistics and comparisons between the male and female respondents in our sample.

Table 1 shows that the male keepers in our sample are significantly older than the female keepers (t = 2.75, *p* = 0.007, df = 93). The male keepers also had more years of playing experience in general (t = 2.36, *p* = 0.020, df = 93) and at a professional level in particular (t = 2.85, *p* = 0.005, df = 93). No significant differences were observed between the groups in terms of the number of respondents who participated in international matches (χ^2^ = 0.62, *p* = 0.431, df = 1), in EHF Champions League (χ^2^ = 0.69, *p* = 0.403, df = 1), played for a national team (χ^2^ = 0.00, *p* = 0.964, df = 1), took part in European or other continental championship (χ^2^ = 1.96, *p* = 0.161, df = 1), at the World Handball Championship (χ^2^ = 0.44, *p* = 0.504, df = 1).

Table 2 presents the number of weekly training sessions in which empty-goal situations were practiced.

We used a chi-square test to verify that there were no significant or near-significant differences between men and women regarding the amount of weekly practice; the obtained result was (χ^2^ = 3.49, *p* = 0.479, df = 4), (χ^2^ = 2.73, *p* = 0.603, df = 4), and (χ^2^ = 3.38, *p* = 0.496, df = 4) for the first, second, and third practice questions, respectively. Table 2 shows that few goalkeepers practiced an empty-goal situation more than twice a week. For further analysis, we used the average for the three practice questions and reduced the number of categories down to three (Table 3): no practice (average = 0), n = 19 (20.0%) goalkeepers; once a week (average between 0 to 1.99), n = 50 (52.6%) goalkeepers; twice or more per week (average larger than 2), n = 26 (27.4%) goalkeepers. Once again, we conducted a chi-square test to identify gender-based differences in the new practice variable (χ^2^ = 0.12, *p* = 0.939, df = 2). Additionally, we tested whether participation in a national team is related to the number of weekly practices of empty-goal scenarios and found no significant correlation (χ^2^ = 2.03, *p* = 0.362, df = 2).

### 3.2. Confidence, Anxiety, Sense of Belonging, and Overall Opinion

We continue by presenting the degree of confidence exhibited by male and female goalkeepers regarding various abilities that are essential while playing empty goal.

A two-way ANOVA was conducted to evaluate the effect of gender and practice on the average of the 10 confidence items that are presented in Table 3. Male keepers (7.41) seem to be more confident than female keepers (6.97); however, this difference failed to reach significance (f = 1.88, *p* = 0.173, df = 1). The amount of practice was not found to significantly contribute to the keepers’ confidence level (f = 0.18, *p* = 0.832, df = 2), and there was no interaction between gender and practice (f = 0.134, *p* = 0.875, df = 2).

A two-way MANOVA was used to explore the effect of gender, practice, and interaction between these two factors on the confidence of the respondents on each of the 10 items (see Appendix A). No significant differences were observed between male and female keepers across the 10 confidence questions (f = 0.53, *p* = 0.864, df = 10, 80). The practice factor was found to be near-significant (f = 1.56, *p* = 0.067, df = 20, 160). We can see in Appendix A that keepers (both women and men) who practice empty-goal scenarios at least twice a week exhibited a higher level of confidence regarding their ability to maintain momentum despite frequent empty-goal substitutions (f = 4.23, *p* = 0.018, df = 2). No significant interaction between gender and practice was detected (f = 1.42, *p* = 0.120, df = 20, 160). One item, however, was shown to have significant interaction; we found that male keepers’ confidence regarding their ability to save a long-distance shot was positively correlated with the number of weekly practices of empty-goal scenarios, whereas female keepers exhibited a negative correlation (f = 3.86, *p* = 0.025, df = 2).

The effect of participation in a national team on the respondents’ confidence was evaluated through a separate two-way MANOVA. Participation in a national team was shown to be a non-significant factor (f = 0.73, *p* = 0.693, df = 10, 82), gender was shown to be non-significant (f = 0.74, *p* = 0.676, df = 10, 82), and the interaction between gender and national team participation was shown to be non-significant (f = 0.73, *p* = 0.689, df = 10, 82).

The goalkeepers’ responses regarding their anxiety level in an empty-goal situation were analyzed in a similar manner.

A two-way ANOVA was conducted to evaluate the effect of gender and practice on the average of the nine anxiety items that are presented at Table 4. We did not find a significant difference in anxiety between women and men (f = 0.81, *p* = 0.368, df = 1), practice did not appear to be correlated with the keepers’ anxiety (f = 0.85, *p* = 0.430, df = 2), and there was no significant interaction between gender and practice (f = 1.97, *p* = 0.144, df = 2).

A two-way MANOVA revealed the difference between female and male keepers across nine anxiety items (see Appendix B) to be non-significant (f = 1.07, *p* = 0.389, df = 9, 81). The effect of practice was found to be significant (f = 2.03, *p* = 0.010, df = 18, 164). For instance, we can see in Appendix B that practice was found to have a significant explanatory power with regard to anxiety concerning the ability of the substitute to arrive at the substitution area on time (f = 3.23, *p* = 0.044, df = 2); respondents with no practice exhibited more anxiety than keepers who participated in weekly practices of empty-goal scenario. The opposite trend emerged in responses regarding the possible damage to personal statistics during empty-goal scenarios; keepers with less practice were found to be less anxious (f = 4.76, *p* = 0.011, df = 2). As for the interaction effect, there was no significant interaction between gender and the amount of practice across the nine anxiety items (f = 0.86, *p* = 0.624, df = 18, 164).

An additional two-way MANOVA was conducted to measure the effect of gender, participation in a national team, and the interaction of these factors on the anxiety exhibited by goalkeepers regarding empty-goal scenarios. Participation in a national team was shown to be a non-significant factor (f = 1.53, *p* = 0.150, df = 9, 83), gender was shown to be non-significant (f = 1.13, *p* = 0.346, df = 9, 83), and the interaction between gender and national team participation was shown to be near-significant (f = 1.79, *p* = 0.081, df = 9, 83).

We proceeded with an examination of goalkeeper responses regarding their sense of belonging within their team during empty-goal scenarios, as well as the keepers’ overall opinions regarding the empty-goal rule.

We used a two-way ANOVA to examine the effect of gender, practice, and the interaction of these factors on the goalkeepers’ sense of belonging and overall approval of the empty-goal rule. Gender was not found to be a significant factor in regard to the keepers’ sense of belonging during empty-goal scenarios (f = 0.23, *p* = 0.627, df = 1), practice was not found to be significant (f = 0.95, *p* = 0.387, df = 2), and the interaction between gender and practice also failed to reach significance (f = 0.16, *p* = 0.850, df = 2). A similar analysis was performed with respect to participation in a national team in the place of the practice variable; none of the variables was found to be significant or near-significant.

As for the general opinion of goalkeepers regarding the empty-goal rule, gender was found to be near-significant (f = 2.86, *p* = 0.094, df = 1), and we can see in Table 5 that the female goalkeepers’ level of approval was 6.10 as compared to the male keepers’ overall opinion of 5.14. Practice was found to be positively correlated with the keepers’ opinion regarding the empty-goal rule (f = 3.56, *p* = 0.032, df = 2), and the interaction between gender and practice was not found to be significant (f = 0.02, *p* = 0.998, df = 2). A similar analysis was performed with participation in a national team in the place of the practice variable; none of the variables was found to be significant or near-significant.

We ran several linear regressions with average confidence, average anxiety, and overall approval of the empty-goal rule as the response variables.

Regressions results shown in Table 6 provided a robustness check of the previously mentioned findings. We can see in Table 6 that male goalkeepers are near-significantly more confident regarding their abilities in an empty-goal situation as compared to female keepers and that the amount of weekly practice is not associated with this confidence (model 1). Participation in a national team did not have any explanatory power with respect to confidence (model 2). These results correspond with the confidence averages presented in Table 3.

A new finding is that more experienced goalkeepers were found to be near-significantly less confident in an empty-goal situation than less experienced goalkeeper (model 1). Additionally, a sense of belonging was found to be a significant predictor of confidence in an empty-goal situation (model 2).

Similar to results in Table 4 regarding the keepers’ level of anxiety, the two regression models did not reveal any significant predictors of anxiety. As for overall opinions regarding the empty-goal rule, the obtained results suggest that less experienced goalkeepers are more positive about the empty-goal rule (near-significant result, model 1), that the amount of weekly practice of empty-goal scenarios is positively correlated with keepers’ approval of this new rule (significant in both models), and that female keepers held a more positive opinion regarding the empty-goal rule as compared to male keepers (model 2). Additionally, we can see that anxiety regarding the empty-goal rule is negatively correlated with approval of the rule (near-significant result, model 2).

Interestingly, we can see in Table 6 that the sense of involvement within the team during empty-goal play is a strong predictor of the keepers’ overall opinion regarding this rule (model 2). We ran several additional models to test whether participation in the World Handball Championship (27 elite-level goalkeepers in our sample) has a unique exploratory power for confidence, anxiety, sense of belonging, or overall approval of the empty-goal rule; none of the models provided significant or near-significant results.

### 3.3. Open-Ended Questions: Advantages and Disadvantages of the Empty Goal Rule

Dozens of the goalkeepers in our sample mentioned the following advantages of the empty-goal rule: Advantage in attack by having one extra player; compensates for one man down due to two-minute suspension; adds to the variety of offensive options and tactical solutions, especially when you struggle to score; and in most cases, raises the offensive efficiency.

Several keepers mentioned that the empty-goal rule can change the pace of the game; the empty-goal tactic can be a good gamble against a stronger opponent when you have nothing to lose.

The following benefits were brought up only once: more penalties and two-minute suspensions for the opposition; requires less effort to create chances for field players on offense and then helps to be more aroused on defense; psychological impact on the defense that is struggling to find a way to stop a team playing seven against six; more exciting and interesting game; physical burden on defense to defend six against seven; the goalkeeper gets an actual chance to score; and sprints can help the goalkeeper stay warm and relieve stress.

As for disadvantages, the following are the keepers’ responses from most to least frequent: risk of easy goals; physical burden on the keeper due to sprints; risk of injury for the keeper; keeper losing concentration while running out and in; the defense is dependent on offense accuracy; risk of two-minute suspension due to illegal substitution; constant mental and physical stress on the substituting player; players forget to substitute; scoring on empty goal is boring for the crowd; goals scored on empty goal demoralize the team; cautious and even hesitant offense; requires a lot of practice; collision between players running to the substitution area; and the two-minute suspension penalty became less meaningful.

## 4. Discussion

We have learned from the literature that sport governing bodies seek to make competitions more attractive to spectators, often by applying changes to sports’ rules. Previous studies (e.g., [1]) have demonstrated that such changes can lead to unexpected and even undesired consequences. In 2016, the International Handball Federation (IHF) introduced a set of rule changes, among which was the empty-goal rule. Several attempts have been made to determine whether attacking with an empty goal is worth the risk. Existing data imply that the efficiency of using the empty-goal tactic depends crucially on timing, ability, and the score [18]. Some studies failed to detect a clear advantage (e.g., [16]); other suggested that playing with an additional field player during the critical periods of handball matches could change the momentum of the game [15].

The goalkeeper is a pivotal figure for implementation of the empty-goal tactic; therefore, in the current study, we examined the empty-goal rule from the goalkeeper’s perspective. Our sample was comprised of 68 male and 27 female professional goalkeepers; the male goalkeepers were older and more experienced within the game of handball than the female keepers (Table 1). However, there were no gender differences in the percentages of keepers who had participated in international matches, EHF champions league, national teams, or continental or World Championships.

We found no difference between female and male goalkeepers in the amount of weekly empty-goal practice (Table 2); additionally, no correlation was recorded between participation in a national team and the amount of practice of empty-goal scenarios. This implies that keepers on higher-level teams who tend to participate in more practice sessions do not benefit from specific preparation for empty-goal situations.

Both female and male goalkeepers exhibited a moderately high level of confidence (around 7 on a 10-point Likert scale) regarding empty-goal-related tasks (Table 3). Practice was shown to have a positive effect on the keepers’ confidence level on only 1 out of 10 items (see Appendix A). Based on regression models (Table 6), we suggest that goalkeepers with more years of experience are less confident with respect to their abilities in empty-goal situations.

As for anxiety, we found no significant effect of gender (Table 4 and Table 6), practice (Table 4 and Table 6), or years of experience (Table 6). Sense of involvement during empty-goal situations was also not found to be a significant predictor of anxiety (Table 6). Overall, the professional goalkeepers in our study exhibited an anxiety level of slightly higher than 3 on a 5-point Likert scale (Table 4). This implies that the goalkeepers may have had some worries or hesitations regarding the empty-goal rule but that they were definitely not stressed-out about it.

Considering the goalkeepers’ overall opinion regarding the empty-goal rule (Table 5), we see that the keepers are not too enthusiastic about the rule; the women’s approval rate was 6.10, and the men’s approval rate was 5.14. Similar to the revealed negative correlation between experience and confidence, the more experienced keepers in our sample were found to be near-significantly more negative regarding the rule (Table 6). This is a rather intuitive result, as goalkeepers with many years of professional experience before the 2016 rule change became used to a more static and predictable style of play. Naturally, such goalkeepers are less willing to change their game in the face of the necessities that the empty-goal rule imposes.

Weekly practice was shown to be positively related to approval of the empty-goal rule (Table 5 and Table 6), which is a positive result from a coaching standpoint. It is important to note here that 24 out of the 95 goalkeepers in our sample reported that an empty-goal situation was not practiced on a weekly basis; 39 out of the 95 keepers stated that it was practiced during only one of their weekly practices. If we take into consideration that a professional handball team has around 10 weekly practices and that, on average, teams receive four 2 min suspensions per match [24], then we realize that the empty-goal tactic is still not being fully integrated into coaches’ practice routine. This is especially true, as it was argued that in situations of numerical inferiority, the empty-goal tactic often constitutes a critical and decisive game moment [25].

Another interesting finding is that the sense of involvement within the team while playing with an empty goal was found to be a significant predictor both for confidence and for approval of the rule (Table 6). The empty-goal rule enables the goalkeeper to extend her/his influence over the offensive end through swift, timely, and coordinated substitutions.

## 5. Conclusions

The most striking result of the current study is that 39% of professional goalkeepers reported that goalkeeper–field player substitution is not a part of their regular training program (see Table 2). This is especially notable, given the importance of the situations in which this tactic is used. Consequently, we urge coaches in general and goalkeeper coaches in particular to put more emphasis on empty-goal scenarios in their training regimes. As opposed to the results of a 2020 survey of elite-level coaches that we mentioned above, our current results leave more room for optimism regarding the empty-goal rule. It seems that young goalkeepers who are trained to adapt to playing empty-goal scenarios may willingly embrace their more dynamic and larger role within the game. The existence of this hypothesized trend is worthy of further examination.

## Figures and Tables

**Table 1:** Means and frequencies for career background questions and comparisons between male and female respondents.

	Total	SD|%	Men	SD|%	Women	SD|%	F|χ^2^	Sig.
Age	28.76	7.89	30.12	8.31	25.33	5.50	7.59	0.007
Years playing	17.40	7.36	18.50	7.95	14.63	4.68	5.59	0.020
Years playing professionally	8.99	6.42	10.13	6.61	6.11	4.94	8.15	0.005
Participated in international matches	80	84.2%	56	82.4%	24	88.9%	0.62	0.431
Participated in EHF Champions League	38	40.0%	29	42.6%	9	33.3%	0.69	0.403
Played for a national team	63	66.3%	45	66.2%	18	66.7%	0.00	0.964
Participated in continental championship	42	44.2%	27	39.7%	15	55.6%	1.96	0.161
Participated in world championship	27	28.4%	18	26.5%	9	33.3%	0.44	0.504
Participated in Olympic Games	6	6.3%	5	7.4%	1	3.7%	NA	NA

SD, standard deviation; F, F test value; Sig, Level of significance.

**Table 2:** Goalkeeper responses regarding the number of weekly practices.

	No Practice	Once	Twice	Three Times	Four Times	Total
We practiced “empty goal” on an entire court, including my substitution	24	39	25	5	2	95
I practiced a run from the substitution area to a shot thrown from a distance	34	30	21	9	1	95
I practiced a substitution with a player, including getting off the bench + a proper substitution	37	27	22	6	3	95

**Table 3:** Means and 95% confidence intervals for female and male goalkeeper responses on perceived-ability questions (10-point Likert scale) divided by the amount of practice of empty-goal scenarios.

Gender	Practice	N	Mean	SE	Lower Bound	Upper Bound
Women	No practice	6	6.96	0.53	5.91	8.02
	Once a week	14	6.97	0.34	6.28	7.66
	Twice or more	7	7.17	0.46	6.26	8.08
	Total	27	6.97	0.26	6.44	7.51
Men	No practice	13	7.28	0.36	6.56	8.00
	Once a week	36	7.32	0.21	6.90	7.75
	Twice or more	19	7.74	0.29	7.16	8.31
	Total	68	7.41	0.17	7.07	7.75

N, number of subjects; SE, standard error.

**Table 4:** Means and 95% confidence intervals for female and male goalkeeper responses on anxiety questions (five-point Likert scale) divided by the amount of practice of empty-goal scenarios.

Gender	Practice	N	Mean	SE	Lower Bound	Upper Bound
Women	No practice	6	3.37	0.32	2.75	4.00
	Once a week	14	3.44	0.21	3.04	3.85
	Twice or more	7	2.81	0.29	2.23	3.39
	Total	27	3.20	0.15	2.89	3.52
Men	No practice	13	3.15	0.21	2.72	3.57
	Once a week	36	2.89	0.13	2.63	3.14
	Twice or more	19	3.08	0.18	2.73	3.43
	Total	68	3.03	0.10	2.83	3.24

N, number of subjects; SE, standard error.

**Table 5:** Means and 95% confidence intervals for the female and male goalkeepers’ sense of belonging and overall opinion divided by the amount of practice of empty-goal scenarios.

	F/M	Practice	N	Mean	SE	Lower Bound	Upper Bound
Degree of belonging and involvement within the team during an empty-goal situation	F	No practice	6	5.83	0.93	3.99	7.68
		Once a week	14	5.93	0.61	4.72	7.14
		Twice or more	7	7.00	0.86	5.29	8.71
		Total	27	6.25	0.47	5.32	7.19
	M	No practice	13	6.15	0.63	4.90	7.41
		Once a week	36	6.53	0.38	5.77	7.28
		Twice or more	19	6.90	0.52	5.86	7.93
		Total	68	6.53	0.30	5.93	7.12
** Overall opinion regarding the empty-goal rule	F	No practice	6	5.00	0.94	3.12	6.88
		Once a week	14	6.29	0.62	5.06	7.51
		Twice or more	7	7.00	0.87	5.26	8.74
		Total	27	6.10	0.48	5.15	7.04
	M	No practice	13	4.00	0.64	2.73	5.28
		Once a week	36	5.36	0.39	4.60	6.13
		Twice or more	19	6.05	0.53	5.00	7.11
		Total	68	5.14	0.31	4.53	5.75

** Significant practice effect, *p* < 0.05; F, female; M, male; N, number of subjects; SE, standard error.

**Table 6:** Multiple linear regression unstandardized coefficients.

	Model 1			Model 2		
	B|R	SE	Sig.	B|R	SE	Sig.
Average confidence						
Gender	0.56	0.30	0.063	0.49	0.30	0.106
Years as a professional	−0.03	0.02	0.074	−0.03	0.02	0.189
Average weekly practice of empty-goal scenarios	0.16	0.19	0.390	0.04	0.14	0.776
Participation in a national team				0.00	0.29	0.987
Sense of belonging				0.13	0.05	0.022
Model R	0.24		0.118	0.33		0.052
**Average anxiety**						
Gender	−0.27	0.18	0.144	−0.28	0.18	0.132
Years as a professional	0.00	0.01	0.934	0.00	0.01	0.685
Average weekly practice of empty-goal scenarios	−0.09	0.11	0.432	−0.06	0.12	0.592
Participation in a national team				−0.20	0.18	0.289
Sense of belonging				−0.02	0.03	0.548
Model R	0.17		0.397	0.22		0.479
**Overall approval of the empty-goal rule**						
Gender	−0.68	0.53	0.206	−1.11	0.47	0.020
Years as a professional	−0.06	0.03	0.089	−0.05	0.03	0.142
Average weekly practice of empty-goal scenarios	1.00	0.33	0.004	0.67	0.29	0.026
Participation in a national team				0.72	0.46	0.124
Average confidence				0.27	0.18	0.145
Average anxiety				−0.54	0.29	0.064
Sense of belonging				0.39	0.09	0.000
Model R	0.37		0.004	0.63		0.000

B, unstandardized regression coefficient; R, the proportion of variance in the dependent variable associated with the predictor variables; SE, standard error; Sig, significance level.

## Data Availability

The data that support the findings of this study are available from the corresponding author upon reasonable request.

## Supplementary Materials

The following are available online at https://www.mdpi.com/article/10.3390/ijerph19116506/s1, Empty-Goal Questionnaire.

## Appendix A

**Table A1:** Responses to 10 confidence items by gender and amount of practice.

	F/M	Practice	N	Mean	SE	Lower Bound	Upper Bound
Make a substitution despite interferences from players on the court	F	No practice	6	7.33	0.95	5.45	9.22
		Once a week	14	6.36	0.62	5.12	7.59
		Twice or more	7	6.71	0.88	4.97	8.46
	M	No practice	13	8.08	0.64	6.80	9.36
		Once a week	36	6.89	0.39	6.12	7.66
		Twice or more	19	7.32	0.53	6.26	8.38
Enter the court according to the rules	F	No practice	6	8.83	0.81	7.23	10.44
		Once a week	14	8.57	0.53	7.52	9.62
		Twice or more	7	7.57	0.75	6.09	9.06
	M	No practice	13	8.46	0.55	7.37	9.55
		Once a week	36	8.64	0.33	7.98	9.29
		Twice or more	19	8.63	0.45	7.73	9.53
Stay aroused between the shots	F	No practice	6	7.50	0.81	5.90	9.10
		Once a week	14	7.43	0.53	6.38	8.48
		Twice or more	7	7.29	0.75	5.80	8.77
	M	No practice	13	7.69	0.55	6.60	8.78
		Once a week	36	7.25	0.33	6.60	7.90
		Twice or more	19	7.26	0.45	6.36	8.16
** Save a long-distance shot	F	No practice	6	8.50	0.72	7.07	9.93
		Once a week	14	7.21	0.47	6.28	8.15
		Twice or more	7	6.43	0.67	5.10	7.76
	M	No practice	13	7.46	0.49	6.49	8.44
		Once a week	36	7.78	0.29	7.19	8.36
		Twice or more	19	8.63	0.41	7.83	9.44
Contribute to offensive success thanks to my substitution	F	No practice	6	7.17	0.89	5.39	8.94
		Once a week	14	7.86	0.58	6.70	9.02
		Twice or more	7	6.43	0.83	4.79	8.07
	M	No practice	13	7.23	0.61	6.03	8.44
		Once a week	36	7.25	0.36	6.53	7.97
		Twice or more	19	7.74	0.50	6.74	8.73
Get up quickly from the bench to a state of fast running	F	No practice	6	7.00	0.90	5.21	8.79
		Once a week	14	7.93	0.59	6.76	9.10
		Twice or more	7	7.29	0.83	5.63	8.94
	M	No practice	13	7.92	0.61	6.71	9.14
		Once a week	36	7.67	0.37	6.94	8.40
		Twice or more	19	7.63	0.51	6.63	8.64
Position myself correctly, on time, in front of the shot	F	No practice	6	6.33	0.81	4.73	7.93
		Once a week	14	6.36	0.53	5.31	7.40
		Twice or more	7	7.57	0.75	6.09	9.05
	M	No practice	13	7.08	0.55	5.99	8.16
		Once a week	36	7.36	0.33	6.71	8.01
		Twice or more	19	8.16	0.45	7.26	9.06
Perform multiple sprints without getting tired	F	No practice	6	5.67	0.91	3.86	7.47
		Once a week	14	5.71	0.60	4.53	6.90
		Twice or more	7	6.57	0.84	4.90	8.24
	M	No practice	13	6.31	0.62	5.08	7.54
		Once a week	36	5.92	0.37	5.18	6.65
		Twice or more	19	6.26	0.51	5.25	7.28
Return to the state of concentration I was in before the substitution	F	No practice	6	5.00	0.82	3.37	6.63
		Once a week	14	6.07	0.54	5.00	7.14
		Twice or more	7	6.57	0.76	5.06	8.08
	M	No practice	13	6.15	0.56	5.05	7.26
		Once a week	36	7.11	0.34	6.45	7.78
		Twice or more	19	6.74	0.46	5.82	7.65
* Maintain the momentum I am in	F	No practice	6	6.33	0.75	4.84	7.83
		Once a week	14	6.21	0.49	5.23	7.20
		Twice or more	7	7.57	0.70	6.19	8.96
	M	No practice	13	6.46	0.51	5.44	7.48
		Once a week	36	7.08	0.31	6.47	7.70
		Twice or more	19	8.32	0.42	7.47	9.16

* Significant practice effect, *p* < 0.05; ** significant interaction effect, *p* < 0.05.

## Appendix B

**Table A2:** Responses to nine anxiety items by gender and amount of practice.

	F/M	Practice	N	Mean	SE	Lower Bound	Upper Bound
** Damaging my success rate (personal statistics)	F	No practice	6	1.83	0.41	1.03	2.64
		Once a week	14	3.29	0.27	2.76	3.81
		Twice or more	7	2.86	0.38	2.11	3.60
	M	No practice	13	2.62	0.28	2.07	3.16
		Once a week	36	2.86	0.17	2.53	3.19
		Twice or more	19	3.32	0.23	2.86	3.77
* A goal scored from a long distance by an opposing player	F	No practice	6	3.83	0.49	2.85	4.82
		Once a week	14	4.14	0.32	3.50	4.79
		Twice or more	7	3.14	0.46	2.23	4.05
	M	No practice	13	3.31	0.34	2.64	3.97
		Once a week	36	3.11	0.20	2.71	3.51
		Twice or more	19	3.16	0.28	2.61	3.71
A goal scored from a long distance by the opposing goalkeeper	F	No practice	6	4.17	0.57	3.03	5.30
		Once a week	14	4.07	0.37	3.33	4.82
		Twice or more	7	3.00	0.53	1.95	4.05
	M	No practice	13	3.62	0.39	2.84	4.39
		Once a week	36	2.97	0.23	2.51	3.44
		Twice or more	19	3.42	0.32	2.78	4.06
The other goalkeeper on my team is more suitable than I am for this style of play, and I am replaced	F	No practice	6	2.17	0.54	1.09	3.25
		Once a week	14	2.14	0.36	1.44	2.85
		Twice or more	7	2.43	0.50	1.43	3.43
	M	No practice	13	2.08	0.37	1.34	2.81
		Once a week	36	2.39	0.22	1.95	2.83
		Twice or more	19	2.37	0.31	1.76	2.98
The coach continues with an “empty goal” even though we received goals	F	No practice	6	4.00	0.52	2.96	5.04
		Once a week	14	3.86	0.34	3.18	4.54
		Twice or more	7	3.57	0.48	2.61	4.53
	M	No practice	13	4.15	0.36	3.45	4.86
		Once a week	36	2.92	0.21	2.49	3.34
		Twice or more	19	3.68	0.29	3.10	4.27
Change in concentration level due to frequent entries and exits to and from the court	F	No practice	6	3.83	0.48	2.87	4.79
		Once a week	14	3.43	0.32	2.80	4.06
		Twice or more	7	2.71	0.45	1.83	3.60
	M	No practice	13	3.54	0.33	2.89	4.19
		Once a week	36	3.11	0.20	2.72	3.50
		Twice or more	19	3.32	0.27	2.78	3.86
Injury (from fast sprints or leaps to the ball)	F	No practice	6	2.83	0.55	1.74	3.93
		Once a week	14	2.93	0.36	2.21	3.64
		Twice or more	7	2.14	0.51	1.13	3.15
	M	No practice	13	3.00	0.37	2.26	3.74
		Once a week	36	2.33	0.22	1.89	2.78
		Twice or more	19	2.53	0.31	1.91	3.14
** Uncertainty about the arrival of my substitute to get to me on time	F	No practice	6	4.67	0.41	3.85	5.48
		Once a week	14	3.93	0.27	3.40	4.46
		Twice or more	7	3.14	0.38	2.39	3.90
	M	No practice	13	3.69	0.28	3.14	4.25
		Once a week	36	3.44	0.17	3.11	3.78
		Twice or more	19	3.53	0.23	3.07	3.98
The reaction of the professional team staff if I do not stop the shot	F	No practice	6	3.00	0.57	1.87	4.14
		Once a week	14	3.21	0.37	2.47	3.96
		Twice or more	7	2.29	0.53	1.24	3.34
	M	No practice	13	2.31	0.39	1.54	3.08
		Once a week	36	2.83	0.23	2.37	3.30
		Twice or more	19	2.42	0.32	1.78	3.06

* Near-significant gender effect, *p* < 0.1; ** significant practice effect, *p* < 0.05.
