# Supplementary material for: The “Empty-Goal” Rule Change from the Perspective of International-Level Team Handball Goalkeepers
Source: Int J Environ Res Public Health. 2022 May 26;19(11):6506. doi: 10.3390/ijerph19116506 (PMC9179990; doi:10.3390/ijerph19116506)
Supplement: Supplementary file 1 [file ijerph-19-06506-s001.zip › ijerph-1706356-supplementary.pdf]

"Empty Goal" Questionnaire

Demographic background:

- Age: ________________
- Gender:
  - male
  - female
- Country of Birth _______________________
- Years of playing handball at any level: _______________
- Years as a professional player: _______________
- Indicate the number of international games that you have participated in:
  - 0
  - 1-10
  - 11-20
  - 21+
- Indicate the number of EHF Champions League games that you have participated in:
  - 0
  - 1-10
  - 11-20
  - 21+
- Indicate the number of appearances for the National team :
  - 0
  - 1-10
  - 11-20
  - 21+
- Participated in European Handball Championship or other continental championship
  - Yes
  - No
- Participated in World Handball Championship
  - Yes
  - No

- Participated in Olympic Games
    - Yes
    - No

Part A

Rate the degree of your confidence in the ability to perform successfully the following on a scale of 1 to 10 as designated below:

	1 Not confidence at all	2	3	4	5	6	7	8	9	10 Highly confidence
Make a substitution despite the interferences from players on the court										
Enter the court according to the rules										
Stay aroused between the shots										
Save a long distance shot										
Contribute to offensive success thanks to my substitution										
Get up quickly from the bench to a state of fast running										
Position myself correctly, on time, in front of the shot										
Perform multiple sprints without getting tired										
Return to the state of concentration I was in before the substitution										
Maintain the momentum I am in										

Part B

Provide 2 benefits/advantages of the “Empty Goal” rule:

1.____

2.____

Provide 2 disadvantages of the “Empty Goal” rule:

1.____

2.____

Part C

Rate your degree of anxiety/worry/frustration regarding the situations described below on a scale of 1 to 5 as designated below:

		1<br>Not anxious at all	2	3	4	5<br>Very anxious
1	Damaging my success rate (personal statistics)					
2	A goal scored from a long distance by an opposite player					
3	A goal scored from a long distance by an opposite goalkeeper					
4	The other goalkeeper on my team is more suitable than I am for this style of play and I am replaced					
5	The coach continues with an “Empty Goal” even though we received goals					
6	Change in concentration level due to frequent entries and exits to and from the court					
7	Injury (from fast sprints or leaps to the ball)					
8	Uncertainty about the arrival of my substitute to me on time					
9	The reaction of the professional team staff if I do not stop the shot					

Part D

Rate the degree of your sense of belonging and involvement within the team during an "Empty Goal" situation on a scale of 1 to 10 as designated below:

1<br>Not involved at all	2	3	4	5	6	7	8	9	10<br>Very involved

Part E

Indicate the number of training sessions per week in which you practice the "Empty Goal" game on a scale of 0 to 4 as designated below:

		0<br>Never	1	2	3	4<br>Four times per week
1	We practice "Empty Goal" on an entire court including my substitution					
2	I practice a run from the substitution area to a shot thrown from a distance					
3	I practice a substitution with a player that includes getting off the bench + a proper substitution					

Part F

Rate your overall opinion regarding the "Empty Goal" rule on a scale of 1 to 10 as designated:

1<br>Highly against	2	3	4	5	6	7	8	9	10<br>Highly in favor
